# Sarcoma with true epithelial differentiation secondary to irradiated glioblastoma 

**DOI:** 10.5414/NP300390

**Published:** 2011-10-18

**Authors:** J. Pimentel, J. Marques, P. Pereira, L. Roque, C. Martins, A. Campos

**Affiliations:** 1 Laboratory of Neuropathology, Department of Neurology, CHLN, EPE- Hospital de Santa Maria,; 2 Department of Neurology, Instituto Português de Oncologia de Lisboa Francisco Gentil,; 3 Cytogenetic Laboratory, CIPM/CEDOC, Instituto Português de Oncologia de Lisboa Francisco Gentil,; 4 Department of Neurosurgery, CHLN, EPE-Hospital de Santa Maria, Lisbon, Portugal

**Keywords:** glioblastoma multiforme, gliosarcoma, fibrosarcoma, epithelial differentiation, radiotherapy

## Abstract

Glioblastoma multiforme rarely shows true, immunohistochemically confirmed, epithelial differentiation. Furthermore, radiotherapy may induce cerebral sarcomatous tumors, and postsurgery glioblastoma irradiation may give rise to secondary gliosarcomas. We report a case of a 48-year-old male operated on a primary glioblastoma, followed by radiotherapy. A local recurrence occurred 23 months later that was operated too, and a second diagnosis of a fibrosarcoma with true epithelial differentiation was made. Primary systemic neoplasms were largely excluded. The patient died shortly after, and postmortem showed another cerebral dural-attached mass corresponding to a sarcoma without epithelial differentiation, and leptomeningeal seeding composed of malignant epithelial elements only. Cytogenetics, however, disclosed the second tumor to be similar to the primary one.

## Introduction 

Glioblastoma multiforme with true epithelial differentiation (TED-GBM), including the presence of squamous cells with epithelial whorls, is one of its rarer subtypes, usually requiring immunohistochemical evidence of both components for diagnosis [1]. However, this differentiation may also be seen in gliosarcomas (GS) [2] and this raises the need for considering the differential diagnosis with metastases of carcinoma and the rare “collision” tumors, in which two histologically distinct tumors temporally and topographically coexist in the nervous system [2, 3, 4]. 

The causal relationship between radiotherapy and nervous system tumors is well known [5, 6], and criteria for radiation-induced tumors are established [7]. Secondary GS may occur either after a radiotherapy-treated GBM [6, 8], or after cranial radiation therapy without a previous history of malignant glioma. Moreover, post-cranial irradiation-related pure sarcomatous tumors have also been described [8]. 

We aim to describe a case of a recurrent tumor in a patient with a GBM submitted to radiotherapy, in which the complete tumor parenchyma had been replaced by an apparent fibrosarcoma (FS) displaying extensive squamous cell differentiation tissue, without postmortem evidence of another systemic tumor. We also try to elaborate on the possible subjacent etiopathogenesis. 

## Case history 

**Figure 1.**
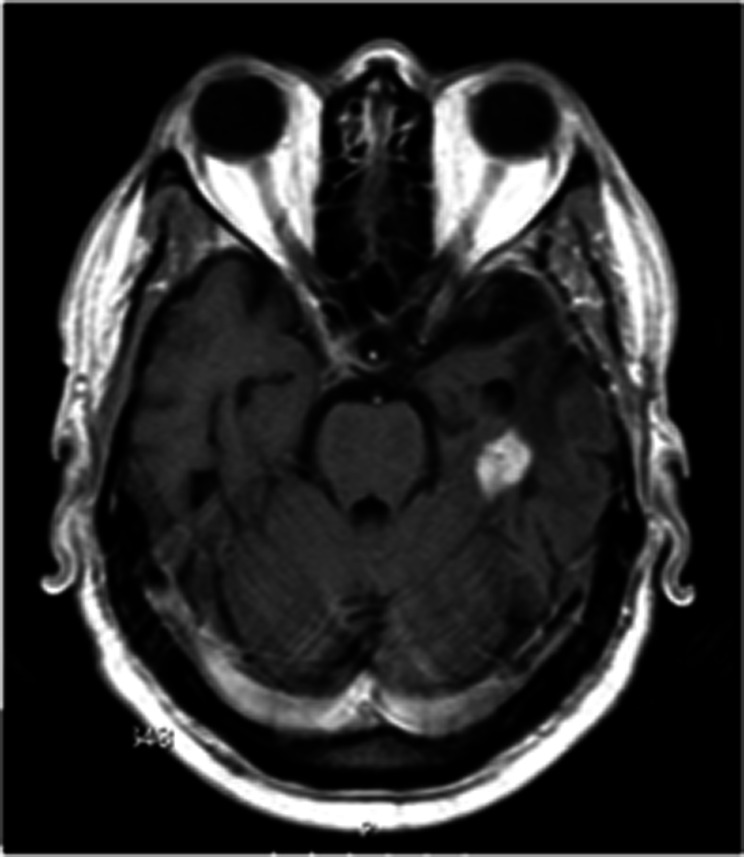
Axial T1 weighted gadolinium enhanced MRI scan showing gadolinium-enhanced nodular lesion in the left temporal lobe.

A 48-year-old male presented with headaches and behavioral changes; the brain MRI (images not available) showed a contrast-enhanced intra-axial mass in the left posterior temporal lobe. A craniotomy was performed with total macroscopic removal of the lesion. After neuropathological diagnosis, the adjuvant treatment consisted of radiotherapy (60 Gy) and chemotherapy with temozolomide. However, 23 months later, a new MRI (Figure 1) disclosed a local recurrence and the patient underwent a second surgery. Following the second neuropathological examination, other primary systemic neoplasms were excluded. A ventriculoperitoneal shunt was placed 4 months later due to an acute hydrocephalus but the patient died of pulmonary thromboembolism. 

## Results 

For the neuropathological study, representative tissues from both surgeries were fixed in 10% buffered formalin and routinely processed for light microscopy. The same procedure was followed for postmortem examination. Slides were stained by hematoxylin and eosin and gomori reticulin. The immunohistochemistry study used the Polimer Dako Envision (Dako, Denmark) as secondary antibody (ab), and the following primary abs: glial fibrillary acidic protein (GFAP, monoclonal ab, 1: 100, Cell Marque/USA), vimentin (monoclonal ab, 1: 100, Novocastra/UK), cytokeratins AE1 AE3 (monoclonal ab, 1: 100, Dako/Denmark) and CK14 (monoclonal ab, 1: 100, Novocastra/UK), epithelial membrane antigen (EMA, monoclonal ab, 1: 100, Cell Marque/USA) and Ki67 (clone MM1, monoclonal ab, 1: 100, Cell Marque/USA) for proliferative evaluation. 

Molecular cytogenetic studies were performed in both tumor samples by high-resolution chromosomal comparative genomic hybridization (HR-CGH) analysis according to a previously described protocol [28]. FISH analyses on tumor imprints were performed with Vysis (Abbott Molecular, Des Plaines, IL, USA) locus specific (LSI) probes: 1p36/1q25; EGFR/centromere of chromosome 7; PTEN/centromere of chromosome 10 and 19q13/19p13 according to the manufacturer’s recommendations. 

**Figure 2.**
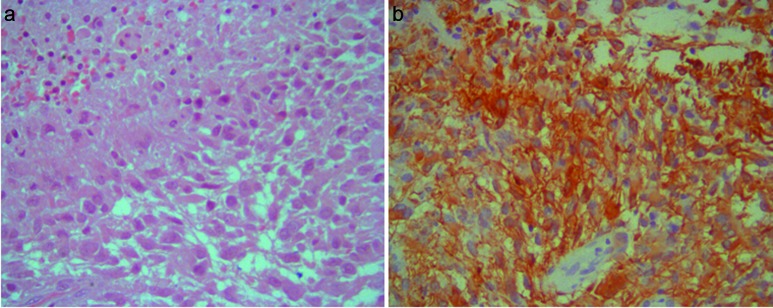
Primitive tumor. a: Neoplastic astrocytes near necrotic tissue (HE × 40); b: GFAP immunoreactive tumor cells (original magnification × 40).

The tumor from the first surgery (Figure 2) was composed of neoplastic, poorly differentiated, pleomorphic, GFAP immunoreactive astrocytes, with nuclear atypia, high mitotic activity, proliferative index higher than 10%, abundant vascular endothelial proliferation and extensive coagulative necrosis. Epithelial markers were all negative. The diagnosis of GBM was made. 

**Figure 3.**
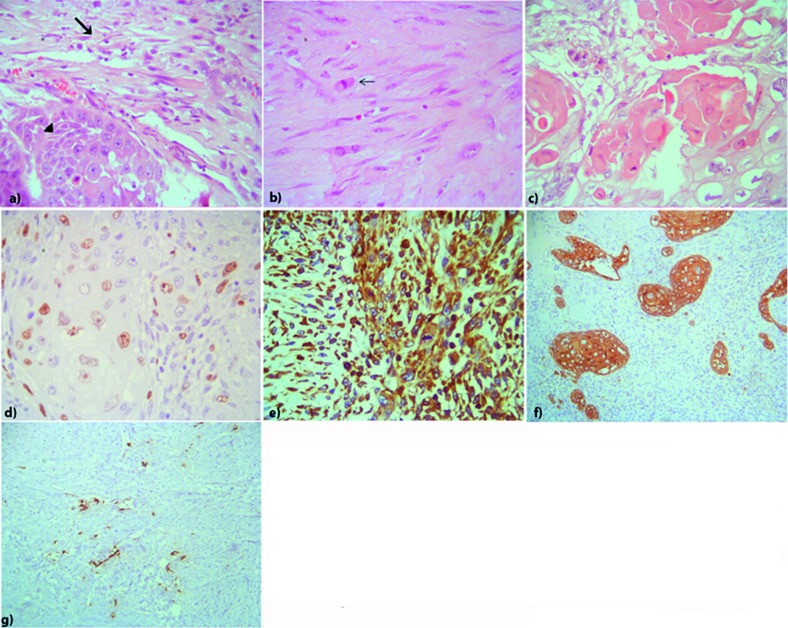
Recurrent tumor. a: Sarcomatous (arrow) and epithelial component (arrow head) (HE, original magnification × 40); b: Sarcomatous component with spindle-shaped, bundle-disposed, malignant elements with mitotic activity (arrow) (HE, original magnification × 40); c: Epithelial component with squamous differentiation and keratin production (HE, original magnification × 40); d: High proliferative index of the epithelial component (Ki67, original magnification × 40); e: Vimentin immunoreactive sarcomatous elements (original magnification × 10); f: Cytokeratin immunoreactive epithelial cells (AE1AE3, original magnification × 10); g: Small nests of GFAP immunoreactive entrapped or reactive astrocytes (original magnification × 10).

The tumor from the second surgery (Figure 3) showed spindle-shaped, bundle-disposed, rich in reticulin, vimentine immunoreactive, malignant elements with moderate mitotic activity, proliferative index higher than 10% and extensive coagulative necrosis. In small islands, sharply separated from the sarcomatous tissue, nests of epithelial cells containing irregularly shaped nuclei with mitotic figures, with squamous differentiation and pearls of keratin were elicited. This epithelial component disclosed immunoreactivity for epithelial markers only (AE1 AE3, CK14 and EMA), and the proliferative index was higher than 30%. Finally, nests of very few, GFAP immunoreactive, astrocytic cells, with no mitotic figures or Ki67 nuclei immunoreactivity, could also be elicited scattered throughout the sarcomatous tissue, although mainly at its periphery. The diagnosis of a FS with TED was made. 

Complex and very similar chromosomal imbalances were observed in both tumors and are described in Table 1. FISH results confirmed HR-CGH data, namely that none of the tumor samples presented EGFR locus amplification. 

General autopsy failed to show any tumor. Macroscopically, there was a right parietal lobe, dural-attached mass, besides left temporal parenchymal changes. 

**Figure 4.**
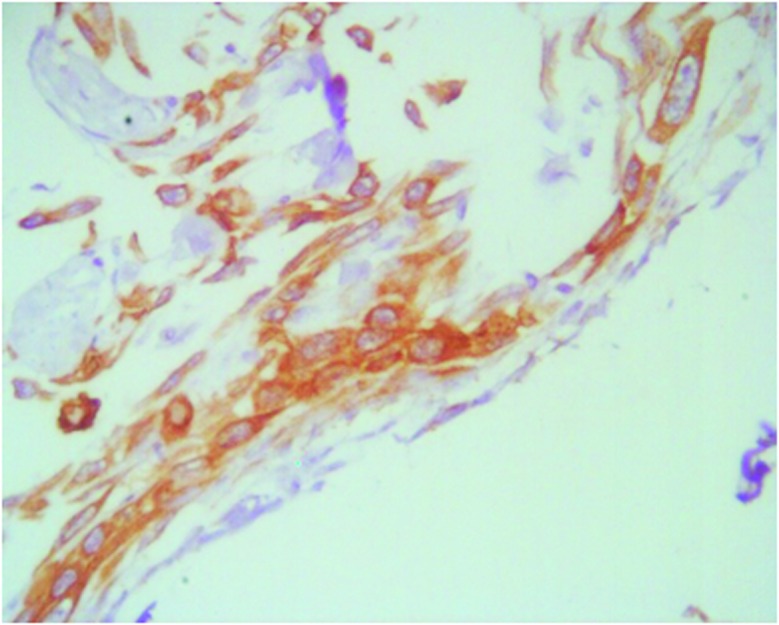
Leptomeningeal, cytokeratin immunoreactive neoplastic cells at the mesencephalic level (AE1AE, original magnification × 40).

Microscopic evaluation (Figure 4) showed leptomeningeal tumor dissemination throughout the neuroaxis by epithelial, AE1 AE3 and CK14 immunoreactive elements only. The parenchymal lesion was composed mainly of coagulative necrosis, with widespread dystrophic microcalcification deposits and reactive astrocytosis, suggesting radiation-related changes. The parenchyma dural-attached mass revealed the same sarcomatous-like tumor as that of the second surgery, but neither epithelial nests nor focal glial cells could be elicited. 

## Discussion 

As mentioned, TED in GBM or GS should be differentiated from epithelioid (epithelial-like cells) and adenoid (compactly arranged cells, occasionally with pseudoglandular/cribiform spaces) GBM, both lacking immunoreactivity for epithelial-specific markers, through immunohistochemical confirmation of this differentiation [1]. Indeed, in the first report of this epithelial-like malignant astrocytic pattern in GS, the presence of transitions from this pattern to neoplastic astrocytes and to their GFAP immunoreactivity is mentioned [9]. A few cases similar to the ones of Kepes et al. were subsequently reported in GBM under different names, all having in common epithelioid, GFAP immunoreactive arrangements of neoplastic astrocytes [1, 10, 11, 12, 13, 14, 15], and several mechanisms were proposed to these distinct tumor cell patterns in GBM and GS [10]. 

TED was first reported by Mørk et al. [2], both in GBM and GS, but this seems to be a very rare occurrence [1, 16, 17], prompting the differential diagnosis with a metastatic carcinoma [9] and the so-called “collision” tumor [2, 3, 4], and with several primary brain tumors [1]. Despite the fact that cytogenetics in our case showed the two apparently different tumors to be the same, in what the association of sarcoma and TED is concerned, the epithelial sarcoma is a well known systemic neoplasia. Two cases of this tumor with cerebral metastases have been described [18, 19] but, from the clinical point of view, they were, as expected, very different from our case. 

Conventional and molecular cytogenetic studies have demonstrated that GBM and GS have a very specific chromosomal profile which is characterized by concurrent trisomy 7 and monosomy 10 with frequent additional gain of chromosomes 1q, 19 and 20, and losses of chromosomes 9 and 22 [20, 21, 22]. Molecular genetic evaluation on TED-GBM or GS [1] has also shown a subset of molecular patterns of GBM with various degrees of epithelial morphology. 

In our case, HR-CGH and FISH studies revealed that the two tumors had very similar cytogenetic alterations, namely, both cases presented +7, +19, +20 and loss of 10. These findings thus point out that the patient’s second neoplasm should be identified as GBM-derived, and exclude the possibility of classifying it as a metastatic carcinoma or a pure FS. The cytogenetic analysis of metastatic carcinomas in the brain has shown that these tumors share the same karyotype alterations of their primary tumor counterparts [23]. FS are a poorly studied group of tumors and, to the best of our knowledge, there are no cytogenetic reports of a primary FS of the brain. In adults, FS analysis revealed that the large majority of these neoplasias presented hypodiploid-diploid chromosome numbers and multiple aberrations, where the only nonrandom chromosome changes described were loss of 9p23-pter and 10q23-qter [24]. Hence, cytogenetics is of paramount importance in situations like the present one, and should be performed routinely [1, 13, 25]. 

The present case has the unique feature of the second operated tumor disclosing a completely different histological type from the first one. Indeed, the possibility of sampling error may exist, although we were cautious enough and studied a quite representative tissue specimen; on the other hand, we are convinced that, given the small islands of Ki67 immunonegative astrocytes spreading throughout the second tumor, this component should be considered entrapped reactive astrocytes and not neoplastic ones. Perry et al. [8] described some cases of GS in which the sarcomatous component grew and dominated the histopathological feature, but it is not mentioned whether they were primary or secondary GS. Given the subarachnoid seeding in postmortem examination, and having found no other tumor in any of the previous MRI, we may speculate that the dural-attached mass should have occurred after radiation, and, accordingly, should be considered a metastasis of the second, FS-like tumor. Regarding the absence of the epithelial component in this dural mass, we could argue that metastases can underexpress the primitive tumor’s full histological pattern. However, we have no explanation for the exclusivity of the epithelial component found in the leptomeningeal seeding. 

The pathogenesis of the aberrant TED in GBM or GS remains unexplained and is poorly addressed in the literature. A hypothetic role of the sarcomatous component in inducing an epithelial pattern was admitted by Kepes et al. [9], but as mentioned, their cases did not concern TED. As quoted by Mørk et al. [2], in experimentally human small-cell glioblastoma transplanted into mice, the presence of abundant connective tissue stroma in the adenoid mucin-producing foci of that tumor was noticed, suggesting the possible inductive role of the mesenchymal component in the development of that aberrant form of differentiation. 

We have no better etiological explanation for the histological differences between both tumors but the previous irradiation. The causal relationship between a brain tumor irradiation and the induction of a neoplasm de novo has been reported for meningioma, cerebral FS and other sarcomatous variants, and, rarely, for GBM [6]. The criteria for radiation-induced tumors are that: it should appear in the area of irradiation; a latency period of years should occur between irradiation and the diagnosis of the tumor; it should have been absent prior to the irradiation; and the tumor de novo should be of a histological type distinct from the previous one [7]. Han et al. [26] reported 30 cases of secondary GS after GBM, 25 having received both external-beam irradiation and chemotherapy and 3 radiotherapy alone, but histopathogical data are mentioned. The same first author had previously reviewed 12 cases of secondary GS and 12 of radiation-induced GS (without previous diagnosis of GBM), comparing clinical and radiological presentation, response to treatment and pathogenesis [6]. Patients underwent a mean irradiation dose of 54.8 Gy, and the mean time from irradiation to GS diagnosis was of 44.8 weeks. The irradiation doses and latency periods were similar and shorter, respectively, for those GS in patients in whom a previous glial component was already present as compared with patients in whom a previous malignant glioma was absent, highlighting the potential role of irradiation in facilitating secondary GS. In our case, the latency period between irradiation and tumor recurrence was more than twice the above mentioned period, suggesting that GS harboring predominantly or exclusively a mesenchymatous component may take longer to develop. Interesting enough and slightly approaching this case to our one, extracranial metastases from a GS in which only the sarcomatous component was detected have been reported [27]. 

The pathogenesis of both forms of secondary GS should be considered similar to the one of primary GS [6]. Moreover, radiation should potentially facilitate the process of: 1) GBM converting local or circulating mesenchymal cells into sarcoma, 2) sarcoma converting local or circulating stem cells into malignant glial cells, or 3) one stem cell lineage giving rise to both malignant glial and mesenchymal elements. Furthermore, radiation could induce a simultaneous genesis of glioma and sarcoma from the same progenitor stem cell, giving rise to a radiation-induced GS [6]. We may speculate that any imbalance in one or more of these mechanisms in the sense of a mesenchymal predominance or exclusivity, potentially genetic in cause, could give rise to a secondary radiation-induced FS-like tumor, instead of a classic GS. 

## Conclusion 

Postsurgery radiotherapy in GBM may increase tumor recurrences histomorphologically very distinct from the primary one, and cytogenetics are of upmost importance in order to diagnose similar or different tumors. The etiopathogenesis of this histomorphological discrepancy is a matter of discussion. When a true epithelial component is added de novo, this fact should prompt more frequently the differential diagnosis with metastases of carcinoma or a collision tumor. FS-like tumor with TED should also be considered in the list of these secondary neoplasias. 

**Table 1.** High resolution – comparative genomic hybridization (HR-CGH) data.

	Chromosomal regions with HR-CGH gains	Chromosomal regions with HR-CGH losses
1^st^ surgery tumor sample	1p32-p31.1, 1p21-p13 1q31, 3q13.1-q13.3, 3q21-q23, 3q26.1-q26.3, 4q12-q24, 5p15.3-p14, 6p22, 7p22-q36, 8p23-p22, 8q22.3-q24.3, 9q22, 9q33-q34, 13q21.3-q31, 16p13.3-p13.1, 17q21-q25, 19q13.2-q13.4, 20p13-13.3, 21p13-21q22.	1q42, 2p16-q21.1, 2q24.3-q37, 3p14-p12, 4p14, 5q14, 6q12-q25, 9p23-p21, 10p14-p12, 10q11.2-q26, 12p13-q24.3, 14q13-q22, 15q14-q24, 16p12, 16q13-q23.
2^nd^ surgery tumor sample	1p32-p31.1, 1p22-p13, 4q13.3-q24, 5p15.3-p15.1, 5q21, 5q23.3-q32, 6p25-p24, 6p22-p21.1, 7p22-q36, 8p23-p21, 8q23-q24.3, 9q21.3, 9q22.3-q33, 13q12-q21.3, 13q31-q32, 16p13.3-p13.1, 17q22-q24, 19q13.2-q13.4, 20p13-13.3, 21p13-21q22.	1q41-q42, 2p13-p12, 2q14.1-q36, 3p14.1-p12, 4p15.3-p12, 6q12-q25, 9p23-p13, 10p15-q26, 12p13-q24.3, 14q11.2-q31, 15q15-q26, 16p12-p11.2, 16q21-q22.

## References

[1] Rodriguez FJ Scheithauer BW Giannini C Bryant SC Jenkins RB Epithelial and pseudoepithelial differentiation in glioblastoma and gliosarcoma: a comparative morphologic and molecular genetic study. Cancer. 2008; 113: 2779–2789 doi:10.1002/cncr.23899 18816605 10.1002/cncr.23899 PMC2597667

[2] Mørk SJ Rubinstein LJ Kepes JJ Perentes E Uphoff DF Patterns of epithelial metaplasia in malignant gliomas. II. Squamous differentiation of epithelial-like formations in gliosarcomas and glioblastomas. J Neuropathol Exp Neurol. 1988; 47: 101–118 doi:10.1097/00005072-198803000-00003 3339369 10.1097/00005072-198803000-00003

[3] Mörk SJ Rubinstein LJ Metastatic carcinoma to glioma: a report of three cases with a critical review of the literature. J Neurol Neurosurg Psychiatry. 1988; 51: 256–259 doi:10.1136/jnnp.51.2.256 2831305 10.1136/jnnp.51.2.256 PMC1031539

[4] Müller W Schröder R Spreading of metastases into cranial tumors: metastasis of a breast carcinoma to a pilocytic astrocytoma. Clin Neuropathol. 1999; 18: 109–112 10361994

[5] Slowik F Balogh I Extracranial spreading of glioblastoma multiforme. Zentralbl Neurochir. 1980; 41: 57–68 6258355

[6] Han SJ Yang I Tihan T Chang SM Parsa AT Secondary gliosarcoma: a review of clinical features and pathological diagnosis. J Neurosurg. 2010; 112: 26–32 doi:10.3171/2009.3.JNS081081 19408981 10.3171/2009.3.JNS081081

[7] Schrantz JL Araoz CA Radiation induced meningeal fibrosarcoma. Arch Pathol. 1972; 93: 26–31 5006996

[8] Perry JR Ang LC Bilbao JM Muller PJ Clinicopathologic features of primary and postirradiation cerebral gliosarcoma. Cancer. 1995; 75: 2910–2918 doi:10.1002/1097-0142(19950615)75:12 < 2910::AID-CNCR2820751219 > 3.0.CO;2-A 7773942 10.1002/1097-0142(19950615)75:12<2910::aid-cncr2820751219>3.0.co;2-a

[9] Kepes JJ Fulling KH Garcia JH The clinical significance of “adenoid” formations of neoplastic astrocytes, imitating metastatic carcinoma, in gliosarcomas. A review of five cases. Clin Neuropathol. 1982; 1: 139–150 6188569

[10] Akimoto J Namatame H Haraoka J Kudo M Epithelioid glioblastoma: a case report. Brain Tumor Pathol. 2005; 22: 21–27 doi:10.1007/s10014-005-0173-6 18095100 10.1007/s10014-005-0173-6

[11] Galloway PG Roessmann U Anaplastic astrocytoma mimicking metastatic carcinoma. Am J Surg Pathol. 1986; 10: 728–732 doi:10.1097/00000478-198610000-00009 3532839 10.1097/00000478-198610000-00009

[12] Kato K Watanabe M Glioblastoma multiforme with epithelial appearance: a case report. Brain Tumor Pathol. 1999; 16: 45–48 doi:10.1007/BF02478901 10532423 10.1007/BF02478901

[13] Kleinschmidt-DeMasters BK Alassiri AH Birks DK Newell KL Moore W Lillehei KO Epithelioid versus rhabdoid glioblastomas are distinguished by monosomy 22 and immunohistochemical expression of INI-1 but not claudin 6. Am J Surg Pathol. 2010; 34: 341–354 doi:10.1097/PAS.0b013e3181ce107b 20118769 10.1097/PAS.0b013e3181ce107b

[14] Mueller W Lass U Herms J Kuchelmeister K Bergmann M von Deimling A Clonal analysis in glioblastoma with epithelial differentiation. Brain Pathol. 2001; 11: 39–43 doi:10.1111/j.1750-3639.2001.tb00379.x 11145202 10.1111/j.1750-3639.2001.tb00379.x PMC8098351

[15] Shintaku M Nakatsu S Okamoto S [“Adenoid” glioblastoma]. No Shinkei Geka. 2000; 28: 359–365 10769835

[16] du Plessis DG Rutherfoord GS Joyce KA Walker C Phenotypic and genotypic characterization of glioblastoma multiforme with epithelial differentiation and adenoid formations. Clin Neuropathol. 2004; 23: 141–148 15328877

[17] Ozolek JA Finkelstein SD Couce ME Gliosarcoma with epithelial differentiation: immunohistochemical and molecular characterization. A case report and review of the literature. Mod Pathol. 2004; 17: 739–745 doi:10.1038/modpathol.3800109 15148503 10.1038/modpathol.3800109

[18] Sugarbaker PH Auda S Webber BL Triche TJ Shapiro E Cook WJ Early distant metastases from epithelioid sarcoma of the hand. Cancer. 1981; 48: 852–855 doi:10.1002/1097-0142(19810801)48:3 < 852::AID-CNCR2820480331 > 3.0.CO;2-J 7248912 10.1002/1097-0142(19810801)48:3<852::aid-cncr2820480331>3.0.co;2-j

[19] Rege AJ Dhir RS Pradeep V Epithelioid sarcoma of the upper extremity with cerebral metastases. J Postgrad Med. 1992; 38: 195–197 1307593

[20] Beroukhim R Getz G Nghiemphu L Barretina J Hsueh T Linhart D Vivanco I Lee JC Huang JH Alexander S Du J Kau T Thomas RK Shah K Soto H Perner S Prensner J Debiasi RM Demichelis F Hatton C Assessing the significance of chromosomal aberrations in cancer: methodology and application to glioma. Proc Natl Acad Sci USA. 2007; 104: 20007–20012 doi:10.1073/pnas.0710052104 18077431 10.1073/pnas.0710052104 PMC2148413

[21] Reis RM Könü-Lebleblicioglu D Lopes JM Kleihues P Ohgaki H Genetic profile of gliosarcomas. Am J Pathol. 2000; 156: 425–432 doi:10.1016/S0002-9440(10)64746-3 10666371 10.1016/S0002-9440(10)64746-3 PMC1850048

[22] Schröck E Thiel G Lozanova T du Manoir S Meffert MC Jauch A Speicher MR Nürnberg P Vogel S Jänisch W Comparative genomic hybridization of human malignant gliomas reveals multiple amplification sites and nonrandom chromosomal gains and losses. Am J Pathol. 1994; 144: 1203–1218 8203461 PMC1887475

[23] Nishizaki T DeVries S Chew K Goodson WH Ljung BM Thor A Waldman FM Genetic alterations in primary breast cancers and their metastases: direct comparison using modified comparative genomic hybridization. Genes Chromosomes Cancer. 1997; 19: 267–272 doi:10.1002/(SICI)1098-2264(199708)19:4 < 267::AID-GCC9 > 3.0.CO;2-V 9258662 10.1002/(sici)1098-2264(199708)19:4<267::aid-gcc9>3.0.co;2-v

[24] Heim S Mitelman F Cancer cytogenetics. Hoboken, N.J.: Wiley-Blackwell 2009;

[25] Nestler U Schmidinger A Schulz C Huegens-Penzel M Gamerdinger UA Koehler A Kuchelmeister KW Glioblastoma simultaneously present with meningioma--report of three cases. Zentralbl Neurochir. 2007; 68: 145–150 doi:10.1055/s-2007-981673 17665342 10.1055/s-2007-981673

[26] Han SJ Yang I Otero JJ Ahn BJ Tihan T McDermott MW Berger MS Chang SM Parsa AT Secondary gliosarcoma after diagnosis of glioblastoma: clinical experience with 30 consecutive patients. J Neurosurg. 2010; 112: 990–996 doi:10.3171/2009.9.JNS09931 19817543 10.3171/2009.9.JNS09931

[27] Weaver D Vandenberg S Park TS Jane JA Selective peripancreatic sarcoma metastases from primary gliosarcoma. Case report. J Neurosurg. 1984; 61: 599–601 doi:10.3171/jns.1984.61.3.0599 6747700 10.3171/jns.1984.61.3.0599

[28] Faria C Miguéns J Antunes JL Barroso C Pimentel J Martins Mdo C Moura-Nunes V Roque L Genetic alerations in a paillary glioneuronal tumor. J Neurosurg Pediatr. 2008; 1: 99–102 18352813 10.3171/PED-08/01/099

